# Senescent HUVECs-secreted exosomes trigger endothelial barrier dysfunction in young endothelial cells

**DOI:** 10.17179/excli2019-1505

**Published:** 2019-09-03

**Authors:** Pooi-Fong Wong, Kind-Leng Tong, Juliana Jamal, Eng-Soon Khor, Siew-Li Lai, Mohd Rais Mustafa

**Affiliations:** 1 Department of Pharmacology, Faculty of Medicine, University of Malaya, Kuala Lumpur, Malaysia

**Keywords:** microvesicular, extracellular vesicle, senescent-associated secretory phenotype (SASP), endothelial adherens junction proteins, senescent HUVECs-secreted exosomes, endothelial barrier dysfunction

## Abstract

Accumulation of senescent endothelial cells can cause endothelium dysfunction which eventually leads to age-related vascular disorders. The senescent-associated secretory phenotype (SASP) cells secrete a plethora of soluble factors that negatively influence the surrounding tissue microenvironment. The present study sought to investigate the effects of exosomes, which are nano-sized extracellular vesicles known for intercellular communications secreted by SASP cells on young endothelial cells. Exosomes were isolated from the condition media of senescent human umbilical vein endothelial cells (HUVECs) and then confirmed by the detection of exosome specific CD63 and CD9 expressions, electron microscopy and acetylcholinesterase assay. The purified exosomes were used to treat young HUVECs. Exposure to exosomes repressed the expression of adherens junction proteins including vascular endothelial (VE)-cadherin and beta-catenin, decreased cell growth kinetics and impaired endothelial migration potential of young endothelial cells. These findings suggest that senescent HUVECs-secreted exosomes could disrupt barrier integrity that underpins endothelial barrier dysfunction in healthy young endothelial cells.

## Graphical Abstract


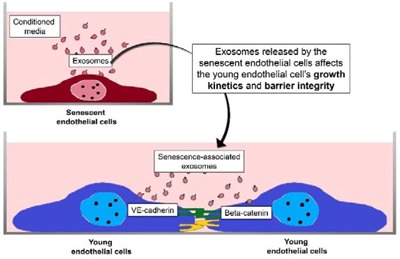


## Introduction

Replicative senescence is a state of irreversible cell cycle arrest in primary cells after 40-60 divisions *in vitro* due to telomeric attrition (von Kobbe, 2018[29]). Premature cellular senescence can also occur in the absence of telomeric attrition in response to excessive extracellular or intracellular stresses including oncogenic mutations, reactive metabolites, DNA damage, proteotoxic stressors as well as high mitogen and nutrient signals which increase mTOR activity (Tchkonia et al., 2013[22]). Senescent cells can accumulate in various human tissues, especially in tissues that experience prolonged inflammation (Bajada et al., 2009[3]; Dimri et al., 1995[11]; Voghel et al., 2007[28]). Although senescent cells are arrested in proliferation, they remain metabolically active, undergo widespread changes in gene and protein expressions and ultimately develop deleterious secretory activity, termed the senescent-associated secretory phenotype (SASP). SASP is known to secrete soluble factors such as inflammatory cytokines (IL-1, IL6), chemokines (IL-8 and monocyte chemoattractant protein-1 (MCP-1)), growth factors (transforming growth factor (TGF), basic fibroblast growth factor (FGF), hepatocyte growth factor (HGF), and vascular endothelial growth factor (VEGF)) and extracellular proteases (matrix metalloproteinases) as a form of communication between the cells (Xu and Tahara, 2013[31]) which plays an imperative role in reinforcing the senescent phenotypes (Kuilman et al., 2008[16]) and promotes pre-cancerous cells proliferation or epithelial-to-mesenchymal transition in susceptible cells (Laberge et al., 2012[17]).

Senescent cells were also reported to secrete nano-sized vesicles termed exosomes (Borghesan et al., 2018[6]; Cocucci et al., 2009[8]; Johnstone et al., 1987[14]). Exosomes are formed within the endosomal compartments and released into the extracellular matrix for intercellular communication (Stoorvogel et al., 2002[21]) and macromolecules transmission between cells (Di Pietro, 2016[9]). Over the past decades, the roles of exosomes in distributing proteins, lipids as well as nucleic acids and taking part in molecular signaling in the development of several diseases have been extensively discussed (Thery, 2011[23]). The presence of exosomes can change tissue microenvironment and initiate inflammation, thrombosis and angiogenesis (Xu and Tahara, 2013[31]). In the development of atherosclerotic plaques, exosomes play a role in the communication between the monocytes and vascular endothelial cells (Camussi et al., 2010[7]). Patients with severe peripheral atherosclerosis as well as with cancers were also reported to have higher peripheral blood exosomes (Anderson et al., 2010[1]). Tumor cell-derived exosomes, termed oncosomes have been reported to be involved in tumor angiogenesis, inflammation, metastasis and drug resistance (Azmi et al., 2013[2]; Di Vizio et al., 2012[10]).

Past research has shown that co-culture of replicative senescent cells with young endothelial monolayer cells could disrupt cell-cell interactions and endothelial barrier integrity (Krouwer et al., 2012[15]). It was also reported that the exosomes of endothelial origin could increase branching formation in vascular development (Sheldon et al., 2010[20]). In the present study, we sought to investigate whether senescent endothelial cells via the secretion of exosomes affect the endothelial barrier function of young cells.

## Materials and Methods

### Establishment of senescent HUVEC cultures by continuous passaging

Human umbilical vein endothelial cells (HUVECs) were purchased from ScienCell Research Laboratories (Carlsbad, CA). Cells were maintained at 37 °C in a humidified atmosphere of 5 % CO_2_ in the air and cultured in endothelial cell medium (ECM) supplemented with 5 % heat-inactivated FBS, 1 % penicillin-streptomycin and 1 % endothelial cell growth supplement (ScienCell Research Laboratories). Senescent HUVECs at passage 22-30 were cultured in conditioned culture medium consisted of ECM supplemented with 5 % exosome-depleted FBS (System Biosciences, United States) and the conditioned medium was recovered for exosomes isolation. Senescence in HUVECs was confirmed by flow cytometry using fluorogenic substrate C_12_FDG to measure senescence-associated-beta-galactosidase (SA-β-gal) activity. Experiments that involved treatment with senescent HUVECs-secreted exosomes were performed using young HUVECs at passage 9-11 cultured in the conditioned culture medium. 

### Isolation of exosomes

Exosomes were isolated using differential ultra-centrifugation as described previously (Thery et al., 2006[24]). The collected culture medium was subjected to successive centrifugations at increasing speed of 300 x g for 10 minutes, 2,000 x g for 10 minutes and 10,000 x g for 30 minutes to eliminate large dead cells and cell debris. The final supernatant was then subjected to ultracentrifugation at 100,000 x g for 90 minutes to sediment small vesicles that correspond to exosomes. The pellet was washed in a large volume of phosphate-buffered saline (PBS) to eliminate contaminating proteins and centrifuged one last time at the same high speed. The exosome pellet was then re-suspended in PBS for further analyses.

### Electron microscopy

Briefly, the frozen isolated senescent HUVECs-secreted exosomes were thawed and mixed with an equal volume of 4 % w/v paraformaldehyde and stored at 4 °C overnight. Carbon-coated Formvar filmed grids were placed on a 10 µl exosome suspension for 30 minutes and washed once with PBS. The exosomes were then fixed in 1 % glutaraldehyde for 5 minutes and washed 7 times with distilled water. The exosomes were stained by dropping 25 % uranyl acetate replacement (UAR) stain for 60 minutes and embedded in a mixture of 0.25 % UAR stain/1.8 % methyl-cellulose for 10 minutes on ice. The exosomes were then air-dried and visualised using transmission electron microscope (TEM) LEO-Libra 120.

### Immunoblotting

Total cellular and exosomal proteins were extracted using RIPA buffer (Santa Cruz, USA) that was supplemented with 1 mM orthovanadate, 2 mM PMSF and 1 % protease inhibitor. The proteins were separated by electrophoresis on 12 % SDS-PAGE and then transferred onto PVDF membranes. The blots were incubated with primary antibodies followed by incubation with HRP-conjugated secondary antibodies. The protein-antibodies complexes were then visualised using the Enhanced Chemiluminescence Western blotting detection system (GE Life Sciences, Amersham). Antibodies against CD63 (H-193) and CD9 (C-4) were purchased from Santa Cruz Biotechnology (USA). Antibodies against VE-cadherin and beta-catenin were purchased from Millipore (Bedford, MA) and Cell Signaling Technology (Danvers, MA), respectively.

### Acetylcholinesterase activity

Acetylcholinesterase activity was assessed as described by Savina et al. (2003[19]). Briefly, 40 μl of the exosome fraction was suspended in 110 μl of PBS. Then, 37.5 μl of this PBS-diluted exosome fraction was added to individual wells on a 96-well flat bottomed microplate. Next, 1.25 mM acetylthiocholine and 0.1 mM 5,5′-dithiobis(2-nitrobenzoic acid) were added to exosome fractions in a final volume of 300 μl, and the change in absorbance at 412 nm was measured every 5 minutes. Data obtained represent acetylcholinesterase enzymatic activity after 30 minutes of incubation.

### Confocal imaging

HUVECs were seeded onto glass coverslips in 12-well plates and incubated for 24 hours in low serum growth medium along with 20 µg/ml or 50 µg/ml of senescent HUVECs-secreted exosomes extracted from senescent HUVECs. After the treatment, the culture medium was removed and cells were fixed with 4 % paraformaldehyde and incubated overnight in the primary antibody. The cells were then washed and treated with secondary Alexa-Fluor 555 and Alexa Fluor 635 conjugated-antibodies (Thermo Fisher Scientific, Waltham, MA), and Hoechst nuclear stain. The coverslips containing cells were then mounted onto slides and images were taken using Leica TCS SP5 II Confocal Imaging System.

### Endothelial cell growth kinetics 

Cell growth kinetics were recorded and measured by transendothelial electrical resistance (TER) using an impedance-based device, ACEA xCELLigence **^®^**Real-Time Cell Analyzer (RTCA) DP (Roche Diagnostics, Mannheim, Germany) as described previously (Bischoff et al., 2016[4]). Briefly, 50 µl of completed culture medium was added to each well of E-plate 16 and the background reading was recorded. A cell suspension of 50 µl at a cell density of 3.0 x 10^3^ cells/well of young HUVECs or 4.5 x 10^3^ cells/well of senescent HUVECs was then added into each well of E-plate 16. Cell attachment and spreading will increase the impedance resistance and the changes of impedance were recorded and expressed as Cell Index (CI) value. HUVECs were allowed to grow in low serum growth medium until confluent. Confluent HUVECs were treated with 20 µg/ml or 50 µg/ml of senescent HUVECs-secreted exosomes extracted from senescent HUVECs. CI value was measured and recorded for another 48 hours. The growth curves were normalized using the CI value of the last measured time point before the addition of senescent HUVECs-secreted exosomes extracted from senescent HUVECs or vehicle control.

### Scratch wound healing assay

Cell migration and cell-cell interaction were examined using scratch wound healing assay. In brief, a cell suspension of 100 µl at a cell density of 8.0 x 10^3^ cells/well of young HUVECs was seeded into each well of 96-well plate. After 24 hours, monolayer culture of young HUVECs was wounded by a horizontal scratched along the center of the well using a 10 µl of the pipette tip. Then, medium containing detached cells was discarded before the treatment with 20 µg/ml or 50 µg/ml of senescent HUVECs-secreted exosomes. Whereas, untreated HUVECs were cultured in ECM supplemented with 5 % exosome-depleted FBS only. Influence of senescent-associated exosomes on wound healing was measured as wound closure or the distance of cell migrated from the edge of the wound after another 24 hours in culture.

### Statistical analysis

Statistical analysis was performed using one-way analysis of variance (ANOVA), followed by Dunnett's multiple comparison post-hoc tests (GraphPad Prism software, version 5.0; GraphPad Software Inc., San Diego, CA). 

## Results

### Establishment of senescent HUVEC cultures

**Figure 1:**
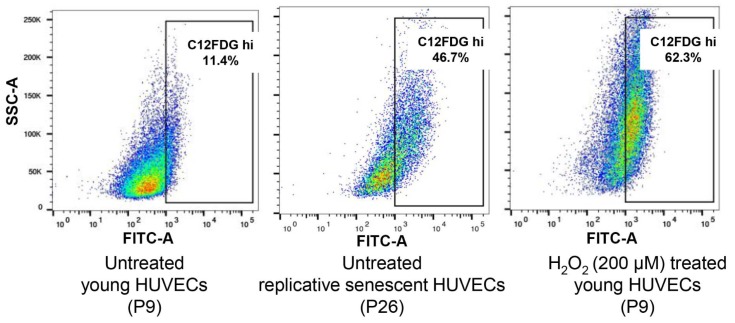
Senescence-associated beta-galactosidase (SA-β-gal) detection in young, replicative senescence (RS) and H_2_O_2_-induced premature senescence. Dot plots represent flow cytometric detection of SA-β-gal in various experimental conditions of HUVECs, young proliferating cells at passage 9, oxidative stress-induced senescent cells following exposures to 200 μM H_2_O_2_ (2 hours exposure per day for 3 days) and replicative senescent cells at passage 26. Cells were pre-treated with bafilomycin A1 to induce lysosomal alkalinization, followed by 1 hour incubation with C_12_FDG, a fluorogenic substrate of β-galactosidase. The gated regions in dot plots of C_12_FDG versus side scatter depict the percentage of cells with bright fluorescence (positive staining of SA-β-gal). Control cells (image not shown) are non-senescent early passage cells.

Senescent HUVECs were established by continuously passaging the cells till near growth arrest. Cellular senescence was confirmed by flow cytometry detection of cells positively stained with SA-β-gal, pH 6.0. Young HUVECs treated with 200 µM H_2_O_2 _were used as control senescent cells. As shown in Figure 1(Fig. 1), the H_2_O_2_-treated cell population contained ~62.3 % senescent cells while young HUVEC culture contained ~11.4 % of senescent cells. Cells that were continuously passaged to establish replicative senescence contained ~46.7 % senescent cells and this showed that senescence was achieved in cell cultures that could be subsequently used for the isolation of exosomes.

### Characterization of extracellular vesicular structures isolated from senescent HUVECs

**Figure 2:**
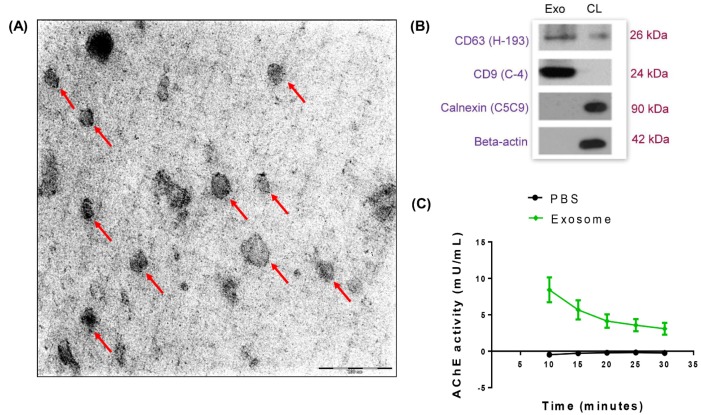
Exosomes in senescent HUVECs conditioned medium. (A) Morphology of exosomes isolated from the conditioned medium of HUVECs was analyzed using transmission electron microscopy. Red arrows indicate exosomes. Scale bar = 200 nm. Magnification = 20,000x. (B) Characterization of exosomes using immunoblotting. An equal amount of exosome fraction (Exo) and HUVECs cell lysate (CL) were analyzed for the expressions of CD63 (H-193), CD9 (C-4), Calnexin (C5C9) and beta-actin. (C) Quantification of acetylcholinesterase (AChE) activity of exosome and PBS was performed.

Extracellular vesicular structures collected from the conditioned media of the senescent cultures were stained with uranyl acetate replacement stain, embedded in methyl-cellulose and then examined by TEM. Numerous extracellular structures with cup-shaped morphology and diameters ranging from 60-100 nm, which are typical of exosomes were observed (Figure 2A(Fig. 2)). Immunoblotting results revealed that the isolated exosomal protein fraction contained a higher abundance of CD63 and CD9 tetraspanin proteins compared to the cell lysates (Figure 2B(Fig. 2)). The absence of calnexin, a chaperone protein of the endoplasmic reticulum and beta-actin in the exosomal protein fraction confirmed no contamination of cellular proteins. These results demonstrated that exosomes were successfully purified from the conditioned culture medium of senescent HUVECs using differential ultra-centrifugation. In addition, the presence of exosomes released was further confirmed by measuring the activity of acetylcholinesterase (AChE), an enzyme that is specific to these microvesicles. AChE activity of the exosomal fraction diluted in PBS was measured compared to PBS alone, after incubation with its substrate, acetylthiocholine. As shown in Figure 2C(Fig. 2), maximal AChE activity of 10 mU/ml was obtained 10 minutes after the initiation of reaction and remained at the level of 5 mU/ml for a duration of 20 minutes in the exosomal fraction (Figure 2C(Fig. 2)). Decreased AChE activity over time was due to the depletion of the substrate, acetylthiocholine throughout the assay. These findings collectively confirmed that extracellular vesicles that were present in the conditioned medium of senescent cell cultures were exosomes.

### Effects of senescent HUVECs-secreted exosomes on endothelial cell-cell junction 

**Figure 3:**
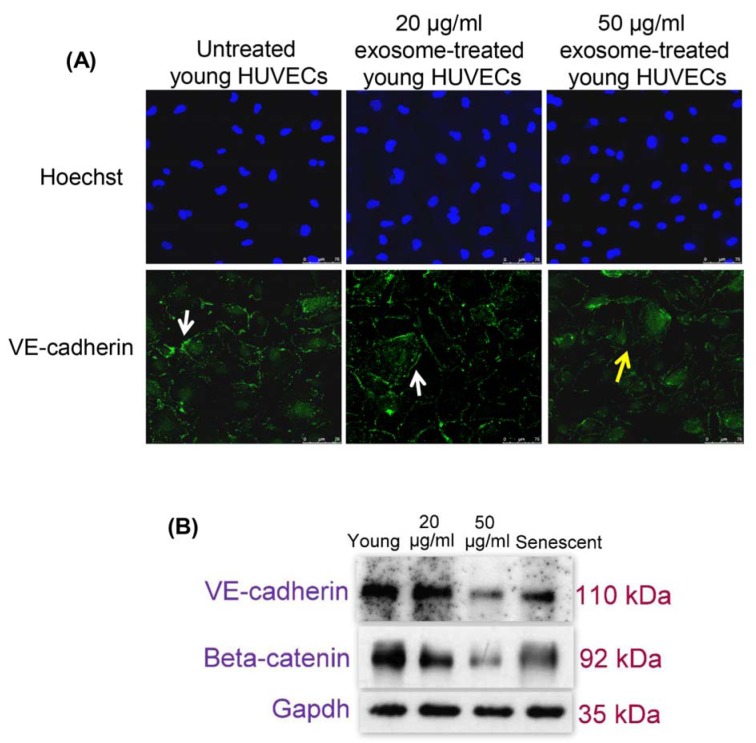
(A) Effects of senescent HUVECs-secreted exosomes on adherens junctions proteins. Young HUVECs were treated with senescent HUVECs-secreted exosomes in ECM supplemented with 5 % exosome-depleted FBS for 24 hours. Immunofluorescent labeling of VE-cadherin in HUVECs. Hoechst staining was used to visualize the nuclei of HUVECs while VE-cadherin stained at cell borders exhibits regular (white solid line arrow) and irregular (yellow solid line arrow) expression pattern. Scale bar = 100 µm. Magnification = 40x. (B) Immunoblotting was performed using total proteins extracted from both untreated and exosome-treated young HUVECs as well as untreated senescent HUVECs to examine VE-cadherin and beta-catenin expression levels. GAPDH served as a loading control.

Immunofluorescence microscopy revealed young HUVECs treated with 50 µg/ml of senescent HUVECs-secreted exosomes contained irregular VE-cadherin disposition inside the cells compared to the normal distribution of VE-cadherin at cell-cell contacts of the untreated young HUVECs and in 20 µg/ml-exosomes treated cells (Figure 3A(Fig. 3)). In addition, the expressions of adherens junction proteins, VE-cadherin and beta-catenin were examined. Immunoblotting analysis showed decreased protein expression levels of VE-cadherin and beta-catenin in senescent HUVECs. Young HUVECs treated with 50 µg/ml of senescent HUVECs-secreted exosomes also had similarly low VE-cadherin and beta-catenin protein expression levels compared to that of the untreated senescent cells (Figure 3B(Fig. 3)). The decreased expression of VE-cadherin following exosomes treatment further corroborated the immunofluorescence findings (Figure 3A(Fig. 3)). These indicated that exposure to senescent HUVECs-secreted exosomes impairs young endothelial cell-cell junction by down-regulating the expression of adherens junction proteins.

### Effects of senescent HUVECs-secreted exosomes on cell growth and proliferation 

**Figure 4:**
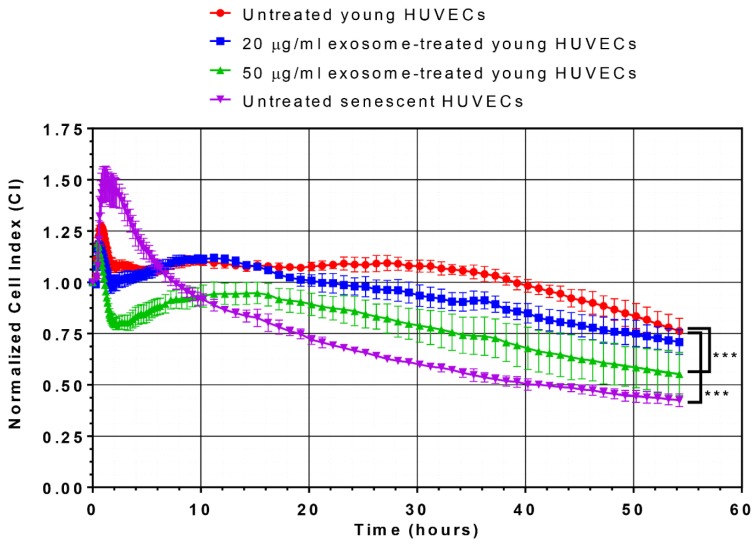
Effects of senescent HUVECs-secreted exosomes on growth kinetic profile. Normalized CI values of HUVECs were monitored for 54 hours in culture. The growth kinetic profile is comprised of normalized CI values of untreated young HUVECs (red line), young HUVECs treated with 20 µg/ml (blue line) and 50 µg/ml (green line) as well as untreated senescent HUVECs (purple line). Statistical significance was analyzed by one-way analysis of variance (ANOVA), followed by Dunnett's multiple comparison post-hoc tests, *** indicates *p *< 0.001 versus the untreated young HUVECs.

As expected, the normalized cell index (CI) values of young proliferating HUVECs were significantly higher than those of the replicative senescence culture. More importantly, a dose-dependent decrease in the normalized CI values in young HUVECs treated with 20 µg/ml and 50 µg/ml of senescent HUVECs-secreted exosomes was observed, whereby, significantly lower CI values were observed in young cells treated with 50 µg/ml of senescent HUVECs-secreted exosomes (Figure 4(Fig. 4)). This finding suggested that senescent HUVECs-secreted exosomes could disrupt the monolayer endothelial cell barrier integrity of young HUVECs and affect its cell growth. 

### Effects of senescent HUVECs-secreted exosomes on cell migration 

**Figure 5:**
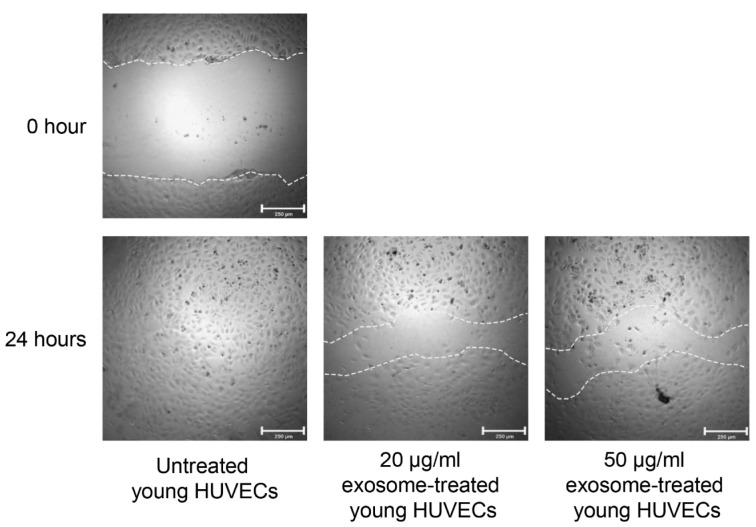
Effects of senescent HUVECs-secreted exosomes on cell migration. Monolayer culture of young HUVECs was wounded by a horizontal scratch along the center of the well using a 10 µl of the pipette tip. The white dotted line drawn at the edge of the wound indicates the wound gap at 0 hour. After 24 hours, cells at the edge of the wound migrated into the wound as shown by shorter distance between the white dotted line. Scale bar = 250 µm.

Endothelial cell migration was examined by *in vitro* endothelial wound healing assay. As shown in Figure 5(Fig. 5), the migration potential of young HUVECs treated with 20 µg/ml and 50 µg/ml of senescent HUVECs-secreted exosomes was impaired (Figure 5(Fig. 5)). Wound closure of the monolayer cultures of HUVECs treated with senescent HUVECs-secreted exosomes was delayed as compared to that of the untreated young HUVECs. After 24 hours of wound induction, untreated young HUVECs showed a complete wound closure as the cells at the edge of the wound has migrated into the wound and closed the wound over time. However, cell migration in young HUVECs treated with both concent-rations of senescent HUVECs-secreted exosomes was impaired resulting in failure of complete wound closure. This implied that senescent HUVECs-secreted exosomes could repress endothelial cell migration.

## Discussion

Exosomes are released by many cell types and known for their roles in intercellular communication. Results from the present study showed that extracellular vesicular structures were present in the conditioned media used to maintain senescent HUVECs cultures. These extracellular vesicular structures were confirmed to be exosomes by the expressions of exosome-specific proteins, CD63 and CD9 tetraspanin proteins and acetylcholinesterase activity.

Impaired cell-cell junction formation, suppressed cell growth and proliferation as well as reduced cell migration can trigger endothelial barrier dysfunction (Zheng et al., 2018[33]). In senescent cells, it is known that adherens junction is disrupted and thus affects endothelial barrier function (Krouwer et al., 2012[15]). Our findings showed in the absence of other soluble factors secreted by senescent cells, exosomes alone at high concentration have a negative impact on the adjoining young endothelial cells as exemplified by reduced cell proliferation, reduced migration potential and disrupted junction morphology with the down-regulation of VE-cadherin and beta-catenin expressions. Previously, it was reported that pre-conditioned medium from senescent cells did not affect VE-cadherin distributions in the neighboring non-senescent cells (Krouwer et al., 2012[15]). In our study, concentrated exosomes purified from pre-conditioned medium of senescent cell culture resulted in relocalization of VE-cadherin proteins from ordered structures lining the adherens junctions to irregular dispositions inside the young neighboring cells and this effect is concentration-dependent. The difference observed between both studies implies that a steady accumulation of soluble factors or exosomes is required to induce pathophysiological changes. This is in agreement with many studies that showed accumulation and persistent presence of a high number of senescent cells as a result of decreased clearance drives the pathophysiological changes associated with aging-related diseases (He and Sharpless, 2017[13]). 

VE-cadherin and beta-catenin are endothelial-specific adhesion molecules located at junctions between endothelial cells (Vestweber, 2008[27]). Beta-catenin links VE-cadherin junction complex to the cytoskeleton to maintain vascular endothelial cell-cell adhesions and barrier function. Cell-cell junctions regulate tissue homeostasis in cellular processes including cell growth and proliferation, and migration (Garcia et al., 2018[12]). Therefore, altered cell-cell junction formation affects signaling mediated through these junctional communications including cell growth and proliferation (Rodrigues and Granger, 2015[18]). Our data demonstrated that senescent HUVECs-secreted exosomes at high concentration could deleteriously influence endothelial cell growth and proliferation. Similarly, wound healing of endothelial monolayer treated with exosomes secreted by senescent cells in our study demonstrated suppression of cell migration which can compromise the ability of the endothelial barrier to repair itself (Zheng et al., 2018[33]). Hence, it is postulated that young endothelial cells respond to the presence of senescent HUVECs-secreted exosomes in the cell culture environment by altering the intercellular junctional communications which leads to the disruption of barrier integrity.

The negative impacts of exosomes-derived from diseased cells are known. For example, oncosomes which are exosomes secreted by tumor cells have been reported to promote angiogenesis, inflammation, tumor metastasis and drug resistance (Azmi et al., 2013[2]). A recent study showed that exosomes released by muscle-derived fibroblasts of Duchenne muscular dystrophy (DMD) patients have a pro-fibrotic role (Zanotti et al., 2018[32]). They further showed that these pro-fibrotic exosomes contain miR-199a-5p that induces trans-differentiation of normal fibroblasts via horizontal transfer (Zanotti et al., 2018[32]). Although miRNA investigation was not performed in the present study, it is highly plausible that these senescent HUVECs-secreted exosomes may contain known vascular inflammation-associated miRNAs or inflamma-miRs, such as miR-19b, -20a, -21, -126, -146a, and -155 as well as miR-221 and-222 that inhibit endothelial cell migration, proliferation and angiogenesis (van Balkom et al., 2013[26]; Urbich et al., 2008[25]). Other reported plausible exosomal-miRNAs include miR-433 that could induce senescence of ovarian cancer cells (Weiner-Gorzel et al., 2015[30]) and miR-143/145 that is responsive to shear stress (Boon and Horrevoets, 2009[5]). Future investigations are warranted to identify miRNAs in senescent HUVECs-secreted exosomes and also whether the horizontal transfer of exosomes containing growth promoting and pro-angiogenic miRNAs could reverse the senescence phenotype change in cells prior to geroconversion. 

## Conclusion

Exosomes are secreted by senescent endothelial cells. Exposure of senescent HUVECs-secreted exosomes to young endothelial cells resulted in decreased expressions of their inter-endothelial adherens junction proteins, VE-cadherin and beta-catenin. This could then lead to altered endothelial junctional communications including suppressed cell growth, proliferation and migration. These results indicate that senescent HUVECs-secreted exosomes could disrupt the endothelial barrier integrity in young healthy cells and compromise endothelial barrier function.

## Acknowledgements

This study was supported by the Fundamental Research Grant Scheme (FP014-2013B).

## Conflict of interest

The authors have no competing interests.

## Authors’ contributions

WPF participated in the whole process of the study; designed the experiment, interpreted the data and prepared the manuscript. TKL, JJ, KES and LSL performed experiments. MRM supervised the project and provided a critical review of the manuscript. All authors read and approved the final manuscript.
